# 高白细胞急性白血病早期死亡的危险因素分析

**DOI:** 10.3760/cma.j.cn121090-20240917-00351

**Published:** 2025-01

**Authors:** 明环 苏, 嶂松 阎, 秋玲 李, 家源 张, 延珂 尹, 波 胡, 永泽 刘, 大鹏 李, 营昌 秘

**Affiliations:** 1 中国医学科学院血液病医院（中国医学科学院血液学研究所），血液与健康全国重点实验室，国家血液系统疾病临床医学研究中心，细胞生态海河实验室，天津 300020 State Key Laboratory of Experimental Hematology, National Clinical Research Center for Blood Diseases, Haihe Laboratory of Cell Ecosystem, Institute of Hematology & Blood Diseases Hospital, Chinese Academy of Medical Sciences & Peking Union Medical College, Tianjin 300020, China; 2 天津医学健康研究院，天津 301600 Tianjin Institutes of Health Science, Tianjin 301600, China

**Keywords:** 高白细胞急性白血病, 白细胞淤滞症, 肿瘤溶解综合征, 早期死亡率, 预后, Hyperleukocytic acute leukemia, Leukostasis syndrome, Tumor lysis syndrome, Disseminated intravascular coagulation, Early mortality rate, Prognosis

## Abstract

**目的:**

总结高白细胞急性白血病（HAL）患者的临床特征，分析该类患者早期死亡的危险因素，为判断早期预后提供依据。

**方法:**

回顾性收集2019年7月1日至2021年11月30日就诊于中国医学科学院血液病医院急救中心的211例原发、初治HAL患者的临床资料，分析各指标对早期危险分层及预后的价值。

**结果:**

①34例（16.1％）患者早期死亡，早期死亡组患者WBC、外周血原始幼稚细胞比例、凝血酶原时间（PT）、纤维蛋白原降解产物（FDP）、D-二聚体水平均高于非早期死亡组（*P*值均<0.05）；②高白细胞急性髓系白血病患者的死亡率（20.5％）高于高白细胞急性淋巴细胞白血病患者（9.3％）（*P*<0.05），两组患者的年龄、肌酐、PT、纤维蛋白原（FIB）水平、WBC、乳酸脱氢酶（LDH）、尿酸、血钾、血钙、血磷水平差异均有统计学意义（*P*值均<0.05）；③当WBC界值为255.96×10^9^/L时，预测患者的早期死亡率敏感度为65.6％，特异度为69.0％，WBC水平高于此值患者的死亡率是低于此值患者的5.164倍（*P*值<0.05）；④就诊时患者的年龄、WBC、LDH、尿素、PT、FDP、D-二聚体是影响HAL患者生存的危险因素（*P*值均<0.05）。

**结论:**

HAL病情凶险，早期死亡率高，年龄、WBC、LDH、尿素、PT、FDP、D-二聚体是HAL患者发生早期死亡的危险因素。

## Abstract (en)

**Objective:**

This study analyzed the clinical characteristics and early mortality risk factors in patients with hyperleukocytic acute leukemia（HAL）to provide a basis for predicting early prognosis.

**Methods:**

Data were retrospectively collected from 211 patients with primary HAL who visited the Emergency Center of the Hematology Hospital, Chinese Academy of Medical Sciences, between July 1, 2019 and November 30, 2021. The value of each indicator in early risk stratification and prognosis was analyzed.

**Results:**

The early-death group exhibited higher WBC, peripheral blood immature cell proportions, prothrombin times（PT）, fibrinogen degradation products（FDP）, and D-dimer levels than the non-early death group（*P*<0.05）. Mortality in hyperleukocytic AML（20.5％）was significantly higher than that in hyperleukocytic ALL（9.3％）（*P*<0.05）. There were significant differences in age, creatinine, PT, fibrinogen（FIB）levels, WBC, lactic dehydrogenase（LDH）, uric acid, blood potassium, blood calcium, and blood phosphorus levels between the two groups of patients（*P*<0.05）. A WBC threshold of 255.96×10^9^/L predicted early mortality with 65.6％ sensitivity and 69.0％ specificity, with higher WBC levels associated with a 5.164-fold increased mortality risk（*P*<0.05）. The age, WBC, LDH, urea, PT, FDP and D-dimer of patients at the time of consultation are risk factors affecting the survival of HAL（*P*<0.05）.

**Conclusion:**

HAL is a life-threatening condition with a high early mortality. Age, WBC, LDH, urea, PT, FDP and D-dimer are risk factors for early death in HAL.

高白细胞急性白血病（Hyperleukocytic acute leukemia，HAL）是指外周血WBC>100×10^9^/L的初诊急性白血病患者（非急性早幼粒细胞白血病）。这部分患者临床起病急骤、进展迅速，并且因易发生白细胞淤滞症、肿瘤溶解综合征（TLS）、弥散性血管内凝血（DIC）等并发症，如果处理不及时、不得当，早期死亡率极高，可达20.1％～40％[1]–[2]。因此，HAL被认为是血液病急危重症，需要紧急治疗干预。本研究对我院急救中心接诊的211例初诊HAL患者的临床资料进行分析，总结HAL患者的临床特征，分析该类患者早期死亡的危险因素，为判断早期预后提供依据。

## 病例与方法

1. 研究对象：2019年7月1日至2021年11月30日就诊于我院血液病急救中心的原发、初治HAL（非急性早幼粒细胞白血病）患者，HAL的诊断分型参照WHO 2016与FAB 1985标准。

2. 资料收集及整理：收集HAL患者性别、年龄及WBC、乳酸脱氢酶、肌酐、血钾、血磷、血钙、活化部分凝血活酶时间（APTT）、凝血酶原时间（PT）、纤维蛋白原（FIB）、纤维蛋白原降解产物（FDP）、D-二聚体等检查结果，本研究已取得患者的知情同意。

3. 降白细胞治疗：降白细胞治疗包括羟基脲（2～6 g/d）、阿糖胞苷（100～200 mg/d，持续静脉滴注）或小剂量地塞米松（1.5～5 mg）。根据WBC、HGB和PLT变化调整治疗药物剂量。同时给予别嘌呤醇降尿酸，水化、碱化尿液，成分输血，抗感染等支持治疗。当外周血WBC降至20×10^9^/L左右时，开始诱导化疗。

4. 转归：主要随访患者早期死亡情况。早期死亡定义为接诊后7 d内死亡。

5. 统计学处理：采用SPSS20.0软件进行统计分析。所有数据均进行正态性检验。正态分布的计量资料以均数±标准差表示，两组比较用两独立样本*t*检验；非正态分布的计量资料以*M*（*IQR*）表示，两组比较采用Mann-Whitney *U*检验。计数资料的组间比较采用*χ*^2^检验。受试者工作特征曲线（ROC曲线）确定cut-off值。采用二分类Logistic回归对早期死亡的影响因素进行单因素分析。以*P*<0.05为差异有统计学意义。

## 结果

一、患者特征

纳入病例总数211例，其中男119例，女92例，男女比例约为1.29∶1，中位年龄27岁（3月龄～85岁）。急性髓系白血病（AML，非急性早幼粒细胞白血病）119例（M_1_ 2例，M_2_ 4例，M_4_ 15例，M_5_ 97例，M_7_ 1例，其中M_4_、M_5_约占94％），WBC的中位值为170.0（101.1～550.4）×10^9^/L。急性淋巴细胞白血病（ALL）87例（B-ALL 55例，T-ALL 32例），WBC中位值为246.0（100.8～996.0）×10^9^/L。混合表型急性白血病2例，婴儿白血病2例，未分化型1例。

二、随访结果

211例患者中，174例早期存活（非早期死亡组），34例早期死亡（早期死亡率16.1％），3例失访。其中34例早期死亡患者中，20例于血液病急救中心死亡，10例于我院住院部死亡，4例于外院死亡。早期死亡患者中AML 24例（高白细胞AML的早期死亡率为20.2％），ALL 8例（高白细胞ALL的早期死亡率为9.2％），婴儿急性白血病2例。自来我院就诊至死亡的中位时间为2.71 d。

分析患者早期死亡原因，主要原因为颅内出血13例（38.2％，AML 9例、ALL 4例），呼吸衰竭15例（44.1％，AML 12例、ALL 3例），TLS 5例（14.7％，ALL 4例、AML 1例），心功能衰竭1例（3.0％）。

三、早期死亡组与非早期死亡组患者临床特征比较

早期死亡组患者年龄、WBC、原始幼稚细胞比例、PT、FDP、D-二聚体、尿素水平均高于非早期死亡组（*P*值均<0.05），HGB水平低于非早期死亡组（*P*＝0.041）；两组患者的PLT、LDH、肌酐、尿酸差异均无统计学意义（*P*值均>0.05）（表1）。

**表1:** 高白细胞急性白血病患者早期死亡组与非早期死亡组患者临床特征比较

项目	早期死亡组（34例）	非早期死亡组（174例）	统计量^a^	*P*值
性别（例，男/女）	19/15	98/76	0.002	0.962
年龄（岁，*x*±*s*）	36.95±23.77	28.13±20.98	2.125	0.035
WBC［×10^9^/L，*M*（*IQR*）］	309.45（177.34，385.54）	173.19（121.49，301.01）	−3.514	<0.001
HGB［g/L，*M*（*IQR*）］	74（58，96）	80（65，99）	−2.047	0.041
原始幼稚细胞比例［％，*M*（*IQR*）］	89（83，94）	85（61，93）	−2.144	0.032
PT［s，*M*（*IQR*）］	15.45（14.03，18.35）	14.60（13.50，15.95）	−2.667	0.008
FDP［µg/ml，*M*（*IQR*）］	19.65（7.63，53.27）	8.30（3.35，21.80）	−2.800	0.005
D-二聚体［mg/L，*M*（*IQR*）］	6.67（1.76，18.17）	2.79（1.03，8.24）	−2.636	0.008
PLT［×10^9^/L，*M*（*IQR*）］	36（23，53）	39（23，64）	−1.013	0.311
LDH［U/L，*M*（*IQR*）］	1 580.85（1 055.27，2 519.45）	1 103.60（694.35，1 957.65）	−2.452	0.014
肌酐［mmol/L，*M*（*IQR*）］	69.95（46.33，94.75）	64.20（49.40，83.70）	−1.152	0.249
尿酸［mmol/L，*M*（*IQR*）］	478.5（423.3，646.0）	458.5（334.3，594.7）	−1.155	0.248
尿素［mmol/L，*M*（*IQR*）］	5.61（3.82，7.21）	4.25（3.37，5.69）	−2.560	0.010
血钾［mmol/L，*M*（*IQR*）］	3.75（2.77，4.34）	3.65（3.29，4.04）	−0.160	0.873
血钙［mmol/L，*M*（*IQR*）］	2.25（2.13，2.34）	2.28（2.17，2.37）	−1.181	0.237
血磷［mmol/L，*M*（*IQR*）］	1.16（0.89，1.45）	1.32（1.00，1.55）	−1.381	0.167
APTT［s，*M*（*IQR*）］	28.75（25.17，32.87）	27.30（25.20，29.55）	−1.593	0.111
FIB［g/L，*M*（*IQR*）］	2.73（1.45，4.05）	2.89（2.01，3.65）	−0.284	0.776

**注** PT：凝血酶原时间；FDP：纤维蛋白原降解产物；LDH：乳酸脱氢酶。^a^性别比较采用*χ*^2^检验，检验统计量为*χ*^2^值；年龄比较采用两独立样本*t*检验，检验统计量为*t*值；非正态分布的计量资料，两组比较采用Mann-Whitney *U*检验，检验统计值为*Z*值

四、高白细胞AML组和高白细胞ALL组临床特征比较

高白细胞AML组的早期死亡率、年龄、肌酐、PT、FIB水平均高于高白细胞ALL组（*P*值均<0.05），初诊时WBC、LDH、尿酸、血钾、血钙、血磷水平均低于高白细胞ALL组（*P*值均<0.05）（表2）。

**表2:** 高白细胞急性髓系白血病（AML）与高白细胞急性淋巴细胞白血病（ALL）患者临床特征比较

项目	AML组（119例）	ALL组（87例）	统计量^a^	*P*值
性别（例，男/女）	66/53	51/36	0.204	0.651
早期死亡率（％）	20.5	9.3	4.691	0.033
年龄（岁，*x*±*s*）	39.21±20.43	18.48±16.67	8.006	<0.001
WBC［×10^9^/L，*M*（*IQR*）］	170.29（122.40，276.91）	246.54（130.71，480.56）	−3.170	0.002
LDH［U/L，*M*（*IQR*）］	1079.6（652.1，1862.2）	1227.4（850.7,2554.3）	−2.227	0.026
肌酐［µmol/L，*M*（*IQR*）］	70.3（54.8,88.9）	56.6（40.4,80.9）	−3.232	0.001
尿酸［µmol/L，*M*（*IQR*）］	407.6（299.7，506.14）	564.5（428.4,713.8）	−5.672	<0.001
血钾［mmol/L，*M*（*IQR*）］	3.39（2.91，3.81）	3.89（3.63,4.29）	−6.401	<0.001
血钙［mmol/L，*M*（*IQR*）］	2.22（2.12，2.33）	2.33（2.27，2.42）	−5.583	<0.001
血磷［mmol/L，*M*（*IQR*）］	1.17（0.93，1.49）	1.40（1.11,1.66）	−3.075	0.002
PT［s，*M*（*IQR*）］	15.0（13.8，16.6）	14.3（13.1,15.9）	−2.190	0.028
FIB［g/L，*M*（*IQR*）］	3.18（2.40，4.03）	2.31（1.74,3.20）	−4.295	<0.001

**注** LDH：乳酸脱氢酶；FIB：纤维蛋白原。^a^早期死亡率比较，采用卡方检验，检验统计量为*χ*^2^值；年龄比较采用两独立样本*t*检验，检验统计量为*t*值；非正态分布的计量资料，两组比较采用Mann-Whitney *U*检验，检验统计值为*Z*值

五、HAL患者早期死亡的危险因素

1. 预测HAL患者早期死亡的指标：绘制年龄、WBC、乳酸脱氢酶、肌酐、血钾、血磷、血钙、APTT、PT、FIB、FDP、D-二聚体各项指标预测HAL患者早期死亡的ROC曲线，计算曲线下面积。通过ROC曲线筛选出对预测HAL患者早期死亡有统计学意义的指标，结果详见表3。其中WBC的ROC曲线下面积最大，AUC为0.673，以255.96×10^9^/L为界值点，预测HAL的早期死亡率的敏感度为65.6％，特异度为69.0％；其他指标ROC曲线下面积依次减小。

**表3:** 各指标预测HAL早期死亡的受试者工作特征曲线（ROC曲线）曲线下面积及最佳界值点、敏感度、特异度

项目	曲线下面积	*P*值	界值点	敏感性（％）	特异性（％）
WBC	0.673	0.002	255.96	65.6	69.0
FDP	0.656	0.005	13.15	68.8	63.2
PT	0.648	0.008	16.55	46.9	81.0
D-二聚体	0.647	0.008	3.89	68.8	60.9
尿素	0.643	0.010	5.14	62.5	67.8
LDH	0.636	0.014	1 239.35	68.8	60.9
年龄	0.630	0.020	29	75.0	57.5

**注** HAL：高白细胞急性白血病；FDP：纤维蛋白原降解产物；PT：凝血酶原时间；LDH：乳酸脱氢酶

2. HAL早期死亡危险因素分析：纳入上述筛选的预测HAL患者早期死亡有统计学意义的指标进行单因素分析，结果显示，年龄以29岁为界值，WBC以255.96×10^9^/L为界值，LDH以1243.7 U/L为界值，尿素以5.14 mmol/L为界值，PT以16.55 s为界值，FDP以13.15 µg/ml为界值，D-二聚体以3.89 mg/L为界值，两组死亡率差异均有统计学意义（*P*值约<0.05）（表4）。

**表4:** 高白细胞急性白血病患者早期死亡危险因素的Logistic回归分析结果

因素	单因素分析		
	*P*值	*OR*	95％*CI*
年龄（>29岁/≤29岁）	0.004	3.243	1.462～7.193
WBC（>255.96×10^9^/L/≤255.96×10^9^/L）	<0.001	4.646	2.115～10.207
LDH（>1239.35 U/L/≤1239.35 U/L）	0.001	3.652	1.645～8.110
PLT（>14.5×10^9^/L/≤14.5×10^9^/L）	0.147	4.529	0.588～34.871
尿素（>5.14 mmol/L/≤5.14 mmol/L）	0.001	3.863	1.785～8.360
PT（>16.55 s/≤16.55 s）	<0.001	4.273	1.975～9.244
FDP（>13.15 µg/ml/≤13.15 µg/ml）	0.001	4.125	1.854～9.175
D-二聚体（>3.89 mg/L/≤3.89 mg/L）	0.001	3.741	1.684～8.310

**注** LDH：乳酸脱氢酶；PT：凝血酶原时间；FDP：纤维蛋白原降解产物

## 讨论

HAL是一种并发症多、早期死亡率高，临床预后差的恶性血液肿瘤。文献报道其主要死亡原因为白细胞淤滞、呼吸衰竭、凝血功能障碍继发的颅内出血[3]。研究发现高白细胞AML的白细胞瘀滞由多种因素引起：①由于髓系白血病细胞比容大于淋系白血病细胞和淋巴细胞，且不如成熟粒细胞或淋巴细胞柔韧，易引起小血管机械性阻塞，导致远端低灌注和缺血性损伤[4]–[5]。因此高白细胞AML比高白细胞ALL患者更常发生白细胞淤滞症。大量白血病细胞聚集在肺组织，会加重局部的缺血缺氧，进一步增加感染的机会，使患者易于发生严重的肺部感染。②白血病细胞产生促炎细胞因子，如TNF-α或IL-1β，诱导内皮细胞表达黏附分子如E-/p-选择素、ICAM-1和VCAM，这些分子与白血病细胞上的黏附分子相互作用[5]。③白血病细胞释放基质金属蛋白酶，破坏内皮细胞的完整性，使白血病细胞外渗到组织中，并导致微出血。这些情况发生于中枢神经系统和肺部时，易导致致命性的颅内出血及呼吸衰竭。白细胞瘀滞的死亡率可高达29％[6]–[7]。本组病例早期死亡患者中颅内出血（38.2％）、呼吸衰竭患者（44.1％）占比较高，应与此有关。

HAL在FAB分型中AML-M_4_、M_5_多见，易伴有FLT3-ITD突变、MLL基因重排[3]，[8]–[9]。HAL患者白细胞瘀滞的发生率可高达45％[3]，Novotny等[10]根据临床特征（主要是神经系统和肺部）提出了预测白细胞瘀滞的评分，认为AML-M_4/5_、高白细胞和原始粒细胞水平者发生白细胞瘀滞的可能性更大。本研究中高白细胞AML患者以M_4_及M_5_为最常见的亚型，约占94％，与文献报道一致。目前对发生白细胞瘀滞的白细胞计数仍不明确。Röllig等[4]发现白细胞计数较低的白血病患者也有白细胞瘀滞的临床表现。本研究中单因素Logistic回归分析结果显示，早期死亡组较非早期死亡组WBC水平高（*P*<0.05），以WBC 255.96×10^9^/L为界值，WBC水平较高组的死亡率是较低组的5.164倍；将其纳入单因素分析，结果显示WBC是早期死亡发生的危险因素。本研究的1周内死亡率为16.1％（其中高白细胞AML的早期死亡率为20.5％，高白细胞ALL的早期死亡率为9.3％），早期死亡原因基本与文献报道相符。所以对于高白细胞AML患者早期积极有效地处理并发症极为重要。

出血和血栓事件是HAL患者早期死亡的最常见原因之一，其中颅内出血占很大比例[7]。DIC在HAL中的发生率约30％[3]。DIC是由于凝血系统的过度激活和纤溶亢进而导致的凝血因子和抗凝血因子的失衡。血栓是因为内皮细胞释放高水平的组织因子，通过凝血因子Ⅶ激活外源性凝血途径[11]；同时，抗凝血因子纤溶酶原激活剂活性增加，纤溶酶原激活剂抑制剂水平降低，白血病细胞膜联蛋白Ⅱ表达增加，以及疾病相关的血小板减少，都促进了DIC的发生[5]。DIC主要通过检测凝血功能来评估,包括FIB、FDP等。本研究结果显示，早期死亡组PT、FDP、D-二聚体水平均高于非早期死亡组（*P*<0.05）；早期治疗时根据检测指标，通过输注冷沉淀、血小板、新鲜冷冻血浆，有时可应用肝素来逆转凝血功能障碍[12]。

有文献报道，高LDH水平提示高肿瘤负荷，同时提示TLS发生的可能，高LDH血清水平是早期死亡的独立危险因素[13]–[14]。本研究发现LDH以1 243.7 U/L为界值，LDH水平较高组的死亡率是较低组的3.739倍，在危险因素研究分析中，LDH与发生早期死亡有关，与文献报道相符。

HAL起病急、进展快、预后差、病死率高，因此识别HAL早期死亡的相关高危因素，并及时给予有效的处置至关重要。如何尽快识别白细胞瘀滞并及时给予合适的治疗，减轻其带来的脏器功能损伤，仍是未来需要研究的重点。
